# Quaternized Poly(*N*,*N*′-dimethylaminoethyl methacrylate) Star Nanostructures in the Solution and on the Surface

**DOI:** 10.3390/polym15051260

**Published:** 2023-03-01

**Authors:** Paulina Teper, Anna Celny, Agnieszka Kowalczuk, Barbara Mendrek

**Affiliations:** Centre of Polymer and Carbon Materials, Polish Academy of Sciences, M. Curie-Sklodowskiej 34, 41-819 Zabrze, Poland

**Keywords:** star polymers, quaternized polymers, star polymer nanolayers, antibacterial surfaces, antibacterial activity

## Abstract

Antibacterial polymeric materials are promising in the fight against resistant bacteria strains. Amongst them, cationic macromolecules with quaternary ammonium groups are one of intensively studied, as they interact with the bacterial membranes causing cell death. In this work, we propose to use nanostructures composed of polycations with star topology for the preparation of antibacterial materials. First, star polymers of *N*,*N′*-dimethylaminoethyl methacrylate and hydroxyl-bearing oligo(ethylene glycol) methacrylate P(DMAEMA-co-OEGMA-OH) were quaternized with various bromoalkanes and their solution behavior was studied. It was shown that in water two modes of star nanoparticles were observed, of diameters about 30 nm and up to 125 nm, independently of the quaternizing agent. Separately layers of P(DMAEMA-co-OEGMA-OH) stars were obtained. In this case, the chemical grafting of polymers to the silicon wafers modified with imidazole derivatives was applied, followed by the quaternization of the amino groups of polycations. A comparison of the quaternary reaction in solution and on the surface showed that in the solution it is influenced by the alkyl chain length of the quaternary agent, while on the surface such relationship is not observed. After physico-chemical characterization of the obtained nanolayers, their biocidal activity was tested against two strains of bacteria *E. coli* and *B. subtilis*. The best antibacterial properties exhibited layers quaternized with shorter alkyl bromide, where 100% growth inhibition of *E. coli* and *B. subtilis* after 24 h of contact was observed.

## 1. Introduction

Today, in many areas of life, the sterility of materials in the context of human health and life is extremely important. This has become particularly important in recent years, when the world began to grapple with the pandemic caused by the SARS-CoV-2 coronavirus. There is a noticeable increase in interest in agents that are able to combat life-threatening pathogens such as bacteria and viruses. In addition, the phenomenon of resistance of pathogenic microorganisms to biocides used so far, mainly in the form of antibiotics, forced researchers to find new solutions that ensure the sterility of materials. In this aspect, polymeric materials are being extensively studied. Antibacterial properties exhibit inter alia poly(4-vinylpyridine) [1,2], polyethyleneimine [3,4] and poly(*N*,*N′*-dimethylaminoethyl methacrylate) (PDMAEMA) [5,6,7,8]. In addition, a variety of macromolecules can be modified by an introduction of functional groups in order to induce biocidal activity in them. Such procedure was successfully carried out for polysiloxanes and polyoxazolines, where ammonium moieties were introduced into the structure of polymers [9,10]. Additionally, PDMAEMA, in order to strengthen the antibacterial effect, is often functionalized in the process of quaternization of its amino groups. Recent studies show that such polymers exhibit a high affinity for the negatively charged bacterial cell wall, which further enhances their antibacterial activity [11]. *N*,*N′*-dimethylaminoethyl methacrylate polymers of simple linear topology were quaternized in the solution [6,12,13], in the form of fibers [6,14] or layers on the solid supports [15,16].

Studies of the biocidal properties of linear polymers of *N*,*N′*-dimethylaminoethyl methacrylate (DMAEMA) quaternized with alkyl halides have shown that the antibacterial activity of such polymers depended on the number of carbons of used quaternizing agent. The biocidal activity of the quaternized PDMAEMA in solution against Gram positive (*S. aureus*) and Gram negative (*E. coli*) bacteria increased with the increase of the number of carbons in the alkyl halide (from 1 to 12) [6,16]. When halides with more than 12 carbons were used for quaternization, the antibacterial activity against the aforementioned bacteria decreased [14,16,17]. Roy et al. [14] for example showed that in the case of the linear polymer DMAEMA grafted onto cellulose and quaternized with higher alkyl bromides (C8, C12 and C16), the best biocidal effect was obtained for PDMAEMA quaternized with 1-bromooctane. The conducted studies do not show a clear influence of the length of the alkyl chain on the obtained antibacterial properties of these polymers.

The topology of a polymer very often affects its biological properties. An interesting example are polymers with star architecture—defined as branched macromolecules in which several or many linear homo- or copolymer chains are covalently bound to one central element, called “the core” [18]. Star polymers very often exhibit stronger biological activity than their linear analogs [19,20] and due to the relative ease of synthesis, multifunctionality and their unique intrinsic properties, they find significant interest in potential applications in biology and medicine. Such application of stars is studied mainly in solution, some of this research is already quite advanced, and solving the problems of in vivo research will hopefully lead to successful clinical trials of these nanomedical systems [21]. On the other hand, a very interesting approach, which is an interesting alternative to conventional polymer coatings, is formation of stable polymer layers based on star macromolecules [22,23,24]. This extends the application possibilities of such materials, which after covalent connection with the solid substrate and appropriate functionalization can serve, for example, as stable and effective layers with antibacterial properties.

The increase in the antibacterial effect of the star polymers is probably due to the better availability of their active biocidal groups in a single macromolecule compared to those found in their linear analogue of the same molar mass [1,6,19,25]. The studies of the antibacterial effect of stars were conducted in different conditions. Liu et al. [6] studied the biocidal effect of the PDMAEMA stars quaternized with alkyl halides of various alkyl chain lengths (from C1 to C12). The tests were carried out in solution and in the form of nanofibers against *E. coli* bacteria. The best antibacterial effect was obtained in the PDMAEMA star solution with amino groups quaternized with heptyl iodide. However, when stars in the form of nanofibers were used, the best antibacterial activity (99% of bacterial cells killed) was observed for 1-iododecane and 1-iodododecane [6]. Similar results were obtained for PDMAEMA grafted onto poly(4-vinylbenzyl chloride) microspheres [16]. These particles resemble star polymers with their topological structure. The authors observed that the increase in methylene groups, from 6 to 12 in the alkyl halide used for quaternization, caused the increase in the bactericidal efficiency of this polymer against *E. coli* and *S. aureus* [16].

In our previous studies, we showed that the both linear and star PDMAEMA in solution [26] and covalently attached to the solid support via photochemical reactions [19] exhibited antibacterial properties both in non-quaternized and quaternized forms. The antibacterial activity of DMAEMA polymers quaternized in the solution with bromoethane, was higher for macromolecules with star topology in comparison with linear, for both Gram-negative (*E. coli* and *P. aeruginosa*) and Gram-positive (*B. subtilis*) bacterial strains. The minimum inhibitory concentration (MIC) and minimum biocidal concentration (MBC) values for quaternized stars were two times lower than quaternary linear polymers for all types of bacteria [26]. In the case of star PDMAEMA layers modified with bromoethane, the best antibacterial properties were obtained in a very short contact (5 min) with *Bacillus subtilis* bacteria. The longer-term antibacterial activity of these layers (24 h) was inhibited in the process of biofilm formation. Additionally, on quaternized linear PDMAEMA layers, the biofilm formed faster than on star polymer layers [19]. Such fast deactivation of the layers by biofilm formation has significantly reduced their usefulness and forces us to look for new chemical improvements to this process.

Above studies support the thesis that there is a limit to the alkyl chain length above which no effect on biocidal properties is observed, the influence of alkyl chain length of used halide is not clear and dependent upon the form of used polymer (solubilized or immobilized on the solid support). In most cases, however, the hydrophobicity of the quaternary agent seems to be the important factor in antibacterial properties, since it controls interaction between the microbial cells and the polymer.

Herein, we compared the conditions of the quaternization process of star polymers with DMAEMA segments in the arms, performed in the solution and for star layers covalently immobilized with the solid supports. First, we synthesized the star polymers with P(DMAEMA-co-OEGMA-OH) arms and quaternized them with bromoethane or 1-bromooctane in the solution. Independently, obtained stars were covalently bonded to silicon wafers using the reaction between the hydroxyl groups coming from OEGMA-OH segments in the arms of the star with an appropriately modified solid substrate. In the next step, the amino groups of the PDMAEMA segments in the form of layers were functionalized using quaternary agents previously applied in the solution. Both the quaternized star polymers in the solution and their layers were characterized by physico-chemical methods. Performed studies allowed us to conclude that modifications of polymeric star nanostructures should be carried out after their attachment to the surface. As layers of such polymers can be used to achieve sterility in many types of materials, we also conducted preliminary studies of their antimicrobial activity against selected bacterial strains. The effectiveness of the obtained polymer nanolayers (quaternized and non-quaternized) on bacterial strains *Escherichia coli* and *Bacillus subtilis* was determined.

## 2. Materials and Methods

### 2.1. Materials

1,2-Dichlorobenzene (99%), 1,1,4,7,10,10-hexamethyltriethylenetetramine (HMTETA, 97%), copper(I) bromide (CuBr, 99.999%), copper(II) bromide (CuBr_2_, 99%), fluorescein sodium salt (>98%), hexadecyltrimethylammonium bromide (≥99%), p-xylene (≥99%), (3-aminopropyl)triethoxysilane (APTES, 99%), 1-carbonyldiimidazole (CDI, ≥97%), were purchased from Sigma Aldrich and used as received. *N*,*N′*-dimethylaminoethyl methacrylate (DMAEMA, ≥99%) was purchased from Merck (Darmstadt, Germany) and purified by distillation prior to use. 1-Bromooctane (99%) and bromoethane (98%) were purchased from Sigma Aldrich (Poznan, Poland) and used as received. Hydroxyl-bearing oligo(ethylene glycol) methacrylate with an average molar mass of M_n_ = 360 g/mol (OEGMA-OH) was purchased from Sigma Aldrich (Steinheim, Germany) and purified according to the procedure described in [27]. Sample of hyperbranched poly(arylene oxindole) (PArOx) which was used as core of the star and simultaneously as macroinitiator was synthesized in prof. Mario Smet group from Catholic University in Leuven (Belgium). Dowex Marathon MSC ion exchanger was purchased from Sigma Aldrich (Saint Louis, MO, USA) and transformed into H^+^ using 1.6 M HNO_3_. Acetone (99.5%), hydrogen peroxide (H_2_O_2_, 30%), methanol (99.8%), and sulfuric acid (H_2_SO_4_, 95%) were purchased from POCH (Gliwice, Poland) and used as received. Tetrahydrofuran (THF, pure p.a.) was purchased from POCH (Gliwice, Poland) and purified by distillation prior to use. Phosphate buffer saline (PBS) was purchased from PAA Laboratories GmbH (Pasching, Austria). Silicon wafers (p-doped, <100>-oriented, 10–20 Ωˑcm resistivity, thickness of 505–545 μm) were purchased from Cemat Silicon S.A. (Warsaw, Poland) and cut into 10 × 10 mm pieces for modification.

### 2.2. Synthesis of the Quaternized Star Polymers

A star polymer (sample SC) with 28 arms composed of *N*,*N′*-dimethylaminoethyl methacrylate and hydroxyl-bearing oligo(ethylene glycol) methacrylate copolymers (P(DMAEMA-co-OEGMA-OH)) and hyperbranched poly(arylene oxindole) (PArOx) core was synthesized according to the procedure published in [28]. The obtained star copolymer was quaternized with bromoethane (sample SCQ_2_) and 1-bromooctane (sample SCQ_8_). For this purpose, to a solution of star SC (3.6 g, 1.8∙10^−5^ mol) in acetone (200 mL), the appropriate alkyl halide, i.e., bromoethane (2.2 mL, 3.3 g, 3.0∙10^−2^ mol) or 1-bromooctane (5.2 mL, 5.8 g, 3.0∙10^−2^ mol) was added. The applied molar ratio of alkyl halide to amino groups in the polymer was 1.5:1. The reaction was carried out in a round bottom flask in an oil bath at 40 °C. The mixture was left on a magnetic stirrer for 24 h. After this time, the reaction mixture became cloudy and was allowed to settle. Then, the acetone was decanted, and the obtained polymer was washed several times (20 mL of acetone for each wash). Solutions of quaternized polymers in acetone were dialyzed against deionized water for 2 days (regenerated cellulose membrane, MWCO 4000–6000 Da) and lyophilized.

The yield of the quaternization process was determined by ^1^H NMR spectroscopy.

### 2.3. Formation of Layers Composed of Quaternized Star Polymers

Star copolymer layers (sample SC-L) were obtained by covalent immobilization of polymer SC to an appropriately functionalized silicon surface. The formation of layers through the chemical reaction between imidazole derivatives attached to the silicon and hydroxyl groups in star arms was performed according to the procedure described in detail in [22]. Afterwards, obtained silicon wafers with immobilized star copolymers (sample SC-L) were placed in the reactor containing 50 mL of acetone and bromoethane (10 mL, 14.7 g, 1.4∙10^−1^ mol) or 1-bromooctane (10 mL, 11.1 g, 5.8∙10^−2^ mol) was added. The ratio of solvent to alkyl halide was 5:1 *v*/*v*. The reactor with the wafers was left on the magnetic stirrer in an oil bath at 40 °C for 24 h. After that time the samples were expansively rinsed with distilled water to remove unreacted alkyl halide, dried in a vacuum, and stored under an argon atmosphere until use. The obtained quaternized layers were designated in the text as samples SC-LQ_2_ for those quaternized with bromoethane and SC-LQ_8_ for those quaternized with 1-bromooctane. The quaternization process was confirmed by determination of the quantity of quaternary groups introduced into the star polymer structure by UV-Vis spectroscopy using methods described in [29,30]. In brief, the amount of fluorescein bonded to the ammonium groups was measured after the quaternization of polymer layers. For this purpose, the wafers with quaternized polymer were immersed in 1 wt% fluorescein sodium saltwater solution for 10 min. The dye binds only to quaternary ammonium groups. After that, the layers were rinsed several times with water and placed in 0.1% aqueous solution of hexadecyltrimethylammonium bromide (3 mL). The layers were shaken in the solution for 20 min at 300 rpm on an orbital shaker to rinse the fluorescein dye previously bonded to the quaternized groups. In described measurements, it was assumed that one fluorescein molecule corresponded to one quaternary amino group of DMAEMA in star polymer.

### 2.4. Evaluation of Antibacterial Activity of the Star Copolymer Nanolayers

The antimicrobial potential was investigated both for non-quaternized (sample SC-L) and quaternized (samples SC-LQ_2_ and SC-LQ_8_) star polymer nanolayers. Wafers with the following parameters were used for the research: mass of wafers with sample: SC-L–0.13 and 0.17 g; SC-LQ2—0.14 and 0.15 g; SC-LQ6—0.12 and 0.16 g, silicon wafers (control)—between 0.09 and 0.12 g. The average size of wafers: SC-L—1.7 cm × 2 cm, SC-LQ2—1.7 cm × 1.7 cm, SC-LQ6—1.7 cm × 1.7 cm, silicon wafers—1.6 cm × 1.7 cm. Model strains representing different genera and taxonomic groups were selected for the study. Reference strains of *Escherichia coli* ATCC 2522 (its pathogenic varieties are a reason of serious infections in humans) and *Bacillus subtilis* 168 (adopted as a model organism for pathogenic microbes such as Staphylococcus aureus) were used.

The antibacterial activity of star copolymer layers against *E. coli* and *B. subtilis* was determined according to ASTM E2149-13a standard test method with minor modifications. The cultures were maintained at 4 °C on Bacto agar slants (Difco, MP Biomedicals, Eschwege, Germany). Inoculums were prepared by transferring the cells from agar slants in Mueller Hinton Broth (MHB) (HiMedia, Mumbai, Maharashtra, India) and were grown overnight at 37 °C with agitation at 200 rpm. Then, bacterial cells were diluted in 0.3 mM KH_2_PO_4_ buffer (pH 7.2) until an OD_470_ = 0.28, corresponding to 1.5–3.0 × 10^8^ colonies forming units (CFU/mL). Appropriately, dilutions were made to obtain a working solution approximately 3.0 × 10^5^ CFU/mL (for sample SC-L and SC-LQ_8_), 2.0 × 10^8^ CFU/mL (sample SC-LQ_2_ for *E. coli*) and 3.0 × 10^3^ CFU/mL (sample SC-LQ_2_ for *B. subtilis*). In one test, under sterile conditions and by duplicate, the wafers of star polymer nanolayers were placed in 100 mL flasks containing 20 mL of the working solution. As a control, in a separate flask containing an identical volume of the working solution, a similarly sized silicon wafer without polymer nanolayers was placed. The samples were incubated at 37 °C with agitation for 1 h. After serial dilutions, the incubated samples were plated in a Petri dish on agar. The inoculated plates were incubated at 37 °C for 24 h and surviving CFU were counted. Subsequently, the incubation of samples continued until 24 h. CFU were counted following the same procedure as after 1 h incubation.

### 2.5. Methods

Gel permeation chromatography with multiangle laser light scattering detection (GPC-MALLS) was used to determine the molar mass and molar mass distributions of the star copolymer (sample SC). System contained a differential refractive index detector (∆n-2010 RI WGE Dr. Bures, Berlin, Germany) and a multiangle laser light scattering detector (DAWN HELEOS from Wyatt Technologies, Santa Barbara, CA, USA) and following set of columns from Polymer Standards Service (PSS): guard + 100 Å + 1000 Å + 3000 Å. Analysis was performed in DMF containing 5 mmol/L lithium bromide at 45 °C with a nominal flow rate of 1 mL/min. The results were evaluated with ASTRA 5 software (Wyatt Technologies, Santa Barbara, CA, USA). The refractive index increment (*dn/dc*) of the star copolymer (sample SC) was estimated in independent measurement in DMF using a SEC-3010 dn/dc WGE Dr. Bures (Berlin, Germany) differential refractive index detector.

The conversion of DMAEMA monomer was determined using gas chromatography (GC) with p-xylene as the internal standard using a VARIAN 3400 gas chromatograph with a J&W Scientific DB-5 (30 m × 0.32 mm) column.

The content of OEGMA-OH in the star SC and quaternization yield of star SC in the case of reaction conducted directly in the solution was calculated based on the NMR spectroscopy. NMR measurements were performed using a Bruker Ultrashield 600 spectrometer (600 MHz for ^1^H) (Billerica, MA, USA). NMR spectra were recorded in ppm and referenced to the tetramethylsilane (TMS) peak.

Dynamic light scattering (DLS) measurements of stars polymers (sample SC, SCQ_2_ and SCQ_8_) in water solution were performed on a Brookhaven BI-200 goniometer with a digital autocorrelator (BI-9000 AT, Brookhaven Instruments, New York, NY, USA) and vertically polarized laser light (Brookhaven Instruments, New York, NY, USA) operating at 35 mW and λ = 637 nm. The autocorrelation functions were analyzed using the constrained regularized algorithm CONTIN. The measurements were made at a 90° angle at 25 °C. Before measurements, the samples were passed through 0.2 µm membrane filters (Graphic Controls, DIA-Nielsen, Düren, Germany).

Zeta potential measurements of star polymers (samples SC, SCQ_2_ and SCQ_8_) were performed in at least triplicate on a Zetasizer Nano ZS 90 (Malvern Instruments, Malvern, UK) in disposable folded capillary cells.

Cryogenic transmission electron microscopy images (cryo-TEM) of samples SCQ_2_ and SCQ_8_ were obtained using a Tecnai F20 TWIN microscope (FEI Company, OR, USA) equipped with a field emission gun, which operates at an acceleration voltage of 200 kV. Images were recorded on an Eagle 4k HS camera (FEI Company, OR, USA) and processed with TIA software (FEI Company, OR, USA). Specimens were prepared by vitrification of star copolymer solutions on grids with holey carbon film (Quantifoil R 2/2; Quantifoil Micro Tools GmbH, Großlöbichau, Germany). Prior to use, the grids were activated for 15 s in oxygen/argon plasma using a Fischione 1020 plasma cleaner (E.A. Fischione Instruments, Inc., Export, PA, USA). Samples were prepared by applying a droplet (2.1 µL) of star polymer solution (samples SCQ_2_ and for SCQ_8_) to the grid, blotting with filter paper and immediately freezing in liquid ethane using a fully automated Vitrobot Mark IV (FEI Company, OR, USA) blotting device. After preparation, the vitrified specimens were kept under liquid nitrogen until they were inserted into a Gatan 626 cryo-TEM-holder (Gatan Inc., Pleasanton, CA, USA) and analyzed in the TEM at −178 °C.

The morphology of obtained star polymer layer (sample SC-L) was investigated with the use of the atomic force microscopy (AFM) in tapping mode with a MultiMode AFM microscope equipped with a NanoScope 3D controller (Veeco Instruments Inc., New York, NY, USA) and 125 nm single crystal silicon cantilevers (Model TESP, Veeco Instruments Inc.). Measurements in air with different scan sizes (from 500 nm × 500 nm to 10 μm × 10 μm) were performed. Images at different surface points were recorded by NanoScope software. Additionally, the root mean square roughness (RMS) was appointed as the average of several measurements of different cross-sections of the images from a 2 × 2 µm surface area.

The thickness of the star copolymer layer on the silicon wafers (sample SC-L) was examined by ellipsometry. The studies were performed using an SE 850E spectrometer (Sentech, Krailling, Germany), working in the spectral range of 240–2500 nm, using Spectra Ray 3 software. The measurements were performed using variable-angle ellipsometric mode at room temperature. Ellipsometric angles (Ψ and Δ), at every 5°, were collected in the 40–70° angle range. The measurements were performed on dry surfaces using the Cauchy method. The thickness of the individual layer was determined using a multilayer optical model: Si/SiO_2_/APTES/CDI.

The quantity of ammonium groups in the layers (samples SCL-Q_2_ and for SCL-Q_8_) was determined by Specord200 Plus (Analytik Jena, Jena, Germany) spectrophotometer equipped with a JUMO dTRON 308 temperature controller (Analytik Jena). The results were analyzed in the ASpect UV software. Measurements were made at a wavelength of λ = 501 nm. The measurements were made based on a previously designated standard curve.

X-ray photoelectron spectroscopy (XPS) was used to confirm the presence of ammonium groups in star polymer layers (samples SC-L, SCL-Q_2_ and SCL-Q_8_). For this purpose, a PHI5700/660 (Physical Electronics, Chanhassen, Minnesota, USA) spectrometer with 1486.6 eV monochromatic Al-Kα radiation (energy resolution of 0.3 eV) was used.

Contact angle measurements of star polymer layers (samples SC-L, SCL-Q_2_ and SCL-Q_8_) were performed using a CAM101 goniometer (KSV Instruments, Ltd., Helsinki, Finland) for dry surfaces at room temperature. The values of contact angle were an average from three places on the wafer. For each place, 30 images were taken from which the software calculated the average value of the contact angle.

## 3. Results and Discussion

### 3.1. Synthesis and Solution Behavior of Quaternized Star Copolymer

**Scheme 1:**
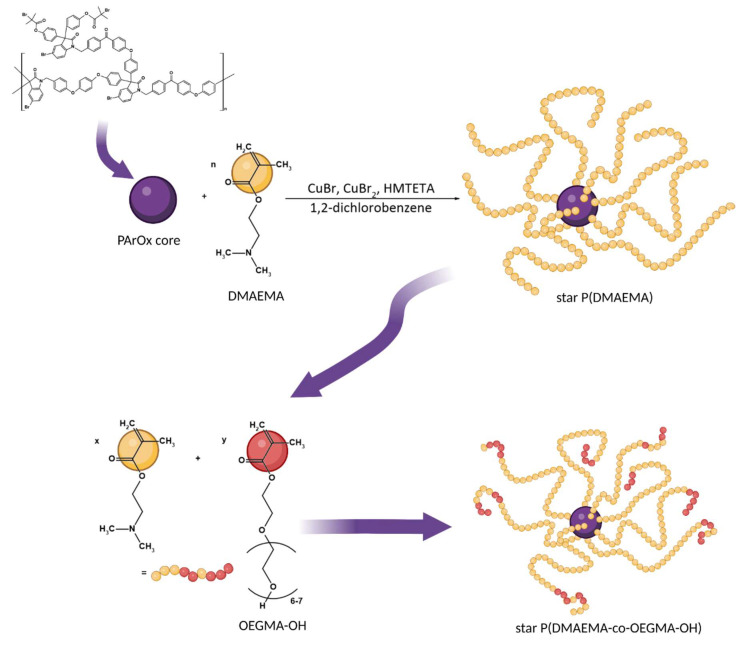
Star copolymer synthesis using ATRP of *N*,*N′*-dimethylaminoethyl methacrylate and hydroxyl-bearing poly[oligo(ethylene glycol) methacrylate] initiated by PArOx.

The star copolymer of *N*,*N′*-dimethylaminoethyl methacrylate and hydroxyl-bearing poly[oligo(ethylene glycol) methacrylate] (P(DMAEMA-co-OEGMA-OH)) was synthesized based on our previously published work [28]. The copolymerization was carried out in such a manner to enrich the ends of the arms in OEGMA-OH units in order to facilitate their subsequent immobilization to the solid substrate. To achieve this, an OEGMA-OH comonomer was added to the same reactor after the appropriate DMAEMA conversion was achieved in so-called “one pot synthesis”. This course of reaction ensured that the OEGMA-OH units were incorporated mainly at the ends of the chains, allowing easy access to the hydroxyl groups in the multi-armed and sterically crowded structure of the star polymer (Scheme 1).

Molar mass and dispersity index (M_w_/M_n_) of obtained star copolymer (SC) was established using GPC-MALLS and was equal 200,000 g/mol and M_w_/M_n_ = 2.5. The value of refractive index increment (*dn*/*dc*) of star P(DMAEMA-co-OEGMA-OH) (sample SC) in DMF was determined in independent measurement and was equal to 0.074 mL/g.

**Figure 1:**
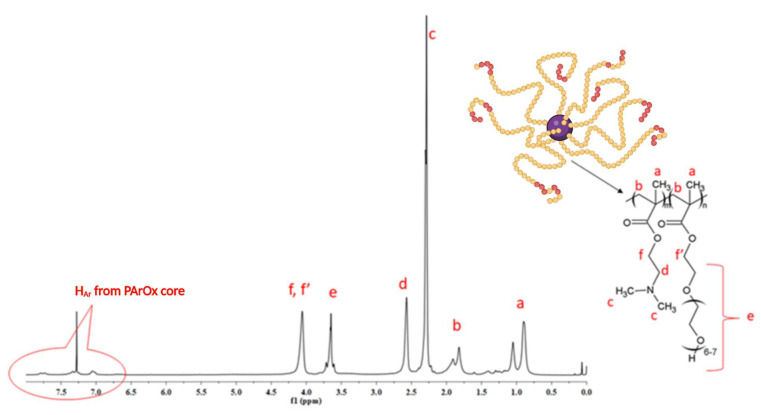
^1^H NMR spectrum of the star copolymer of DMAEMA and OEGMA-OH (CDCl_3_, 600 MHz).

The OEGMA-OH content in the star polymer arms was calculated from ^1^H NMR spectrum in chloroform (Figure 1), as the ratio of the signal of the protons of the methyl groups bound to the amino group in DMAEMA (c) to the signal of the protons of the methylene groups in the OEGMA-OH chains (f) and ranged 7%.

It is known, that after the quaternization of PDMAEMA amino groups, highly effective biocidal polymers were obtained, killing for example such strains of bacteria as *Escherichia Coli* [3,6,15,16,30], *Staphylococcus aureus* [16] or *Bacillus subtilis* [15,31]. In our studies, in the first step, the conditions for the quaternization reaction in solution were established. For this purpose, the obtained star copolymer (sample SC) was reacted with bromoethane and 1-bromooctane, yielding polymers SCQ_2_ and SCQ_8_, respectively.

**Figure 2:**
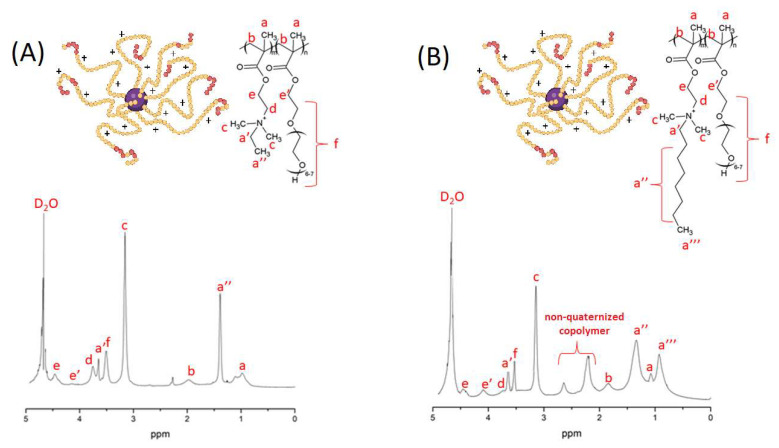
^1^H NMR spectrum of the star copolymer SC after quaternization reaction with: (**A**) bromoethane (sample SCQ_2_) and (**B**) 1-bromooctane (sample SCQ_8_) (D_2_O, 600 MHz).

The yields of the quaternization reactions were calculated from the ^1^H NMR spectra (Figure 2A,B). The both spectra of the star copolymers, quaternized with bromoethane (Figure 2A) and 1-bromooctane (Figure 2B), show shift characteristic of the protons signals of the groups N^+^ -CH_2_- at δ = 3.4 ppm and N^+^ -CH_2_-C_7_H_15_ at δ = 1.3 ppm, which are indicative of an N-alkylation. The yield of reaction with bromoethane was equal to 100%, as no signals from the amino groups of DMAEMA units in the ^1^H NMR spectrum were visible (Figure 2A). In the case of 1-bromooctane, the calculated reaction yield was equal to 60%, as the signals from amino groups were still observed in the ^1^H NMR spectrum (Figure 2B). Extending the reaction time to one week did not affect the yield of the process. The probable reason is the presence of the long alkyl chain of the quaternized agent which had impeded access to amino groups in the arms of the star with a relatively crowded and complex structure. Incomplete reaction efficiency may also be the result of emerging changes in electrostatic interactions between polycationic star chains and introduced long alkyl halide in the studied system. The obtained results indicate that the alkyl chain length has a decisive influence on the efficiency of the star quaternization reaction in acetone solution. Analyzing the efficiency of the reaction in the context of the presence of OEGMA-OH units in the star, which may be unevenly distributed in its structure, it can be concluded that it has no significant impact on the quaternization with bromoethane. Nevertheless, the distribution of OEGMA-OH may have an impact on lowering the efficiency of the reaction with a 1-bromooctane.

**Figure 3:**
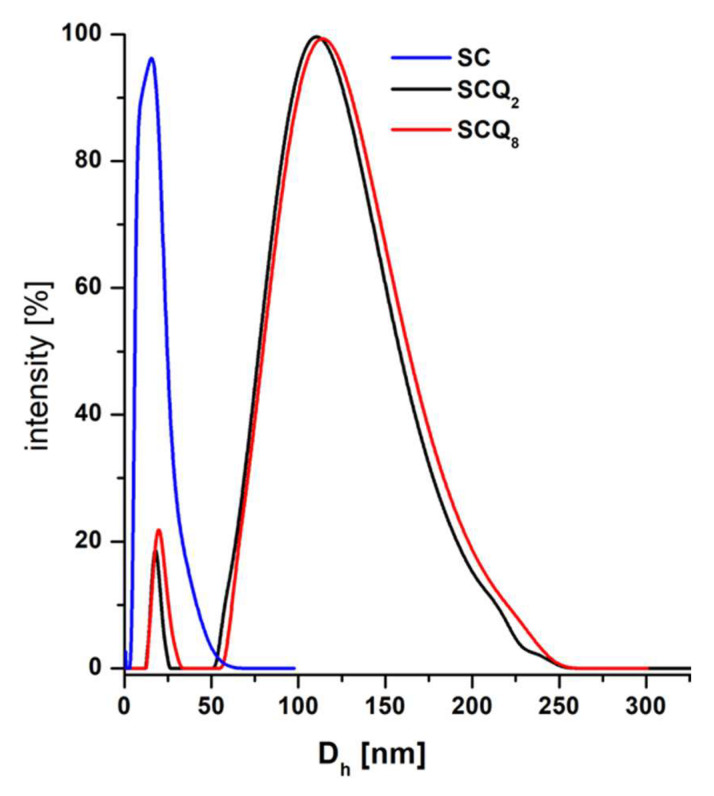
Distributions of the hydrodynamic diameter of P(DMAEMA-co-OEGMA-OH) stars before (sample SC) and after quaternization with bromoethane (sample SCQ_2_) and 1-bromooctane (sample SCQ_8_) in water (c = 1 mg/mL).

Since the size of star polymers is a very important feature that often determines the possibility of their applications in biomedicine and is also a value that allows in some cases to refer to the thickness of the layers obtained from them, the hydrodynamic diameters of the star nanostructures obtained after quaternization were determined in solutions using dynamic light scattering (DLS). Very often in water solutions, branched polymers aggregate to various types of structures and the study of this type of processes is necessary to estimate the possibility of their future bioapplications [32,33,34]. The sizes of the obtained star SC before and after quaternization reactions measured by DLS in water are shown in Figure 3.

The size distribution of non-quaternized star copolymer SC in water at pH of dissolution was asymmetric but monomodal, the hydrodynamic diameter measured in water was equal to 34 nm. The calculations of contour length of the star (with molar mass 200,000 g/mol and 7% of OEGMA-OH which corresponds to a degree of polymerization of 40 per arm) for fully extended arms show that it is equal to 20.16 nm. This value was obtained by multiplying the DP of the two arms and the length of one repeating unit containing two carbon atoms in the backbone equal to 0.252 nm [35]. Comparison of the calculated and determined hydrodynamic diameters indicates that in water solution star polymers self-assemble to small aggregates of a few macromolecules.

Regardless of the type of alkyl bromide used in the quaternization reaction, the nanostructures with significantly higher sizes in comparison to star SC were visible (Figure 3). The hydrodynamic diameters of aggregated structures were equal to D_h_ = 110 nm for SCQ_2_ and D_h_ = 125 nm for SCQ_8_. In both cases, however, a mode corresponding to the smaller size of star nanostructures was also observed (about 20–25 nm). The obtained results indicate that the introduction of ammonium groups promotes additional aggregation of nanostructures in aqueous solution.

For the obtained nanostructures in water solutions, the values of zeta potential, which determines the surface charge of nanoparticles, were also determined. Generally, at a pH close to neutral, PDMAEMA is charged to a limited degree, while after quaternization the chains are positively charged independent of solution pH. The measurements carried out in water in pH of polymer dissolution showed that the value of zeta potential of star polymer SC was equal to 31 ± 1 mV. After the introduction of ammonium groups into the star arms, zeta potential of obtained nanostructures increased significantly, to 62 ± 1 mV for star SCQ_2_ and to 53 ± 1 mV for star SCQ_8_. The lower value of zeta potential, obtained in the case of star SCQ_8_ quaternized with longer alkyl bromide may result from lower yield of the quaternization reaction. Similar increase in zeta potential values after quaternization performed with methyl iodide was observed for miktoarm stars with PDMAEMA and POEGMA arms (from 24.2 mV to 56.4 mV) [36].

**Figure 4:**
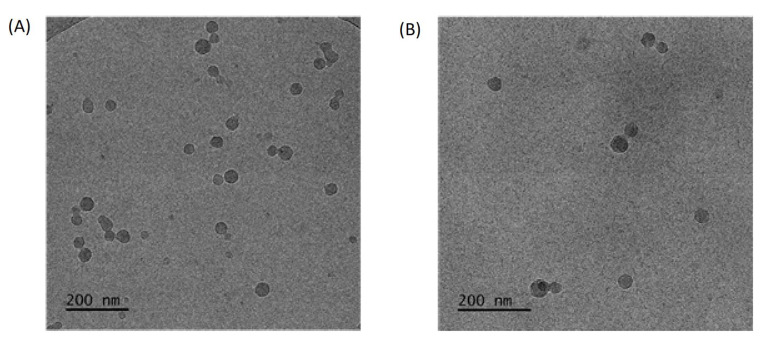
Cryogenic transmission electron microscopy images of P(DMAEMA-co-OEGMA-OH) stars after quaternization (**A**) sample SCQ_2_) (**B**) sample SCQ_8_.

Cryogenic transmission electron microscopy (cryo-TEM) images of the quaternized star polymers (samples SCQ_2_ and SCQ_8_) showed spherical structures, in some cases interconnected into larger clusters (Figure 4). This effect is more pronounced in the case of sample SCQ_8_ where the quaternization degree was lower (60%) (Figure 4B) which in turn could have led to a weakening of the repulsive electrostatic interactions between stars and promoted their aggregation. This confirms the DLS results where larger aggregate sizes were obtained for the sample SCQ_8_.

### 3.2. Formation and Quaternization of Star P(DMAEMA-co-OEGMA-OH) Layers

The quaternization reaction using both bromides was also tested for star polymers covalently bonded to a silicon substrate. To achieve such nanolayers, first the star copolymer SC was immobilized on the support using a so-called “grafting to” method. First, 1-carbonyldiimidazole (CDI) was introduced on silicon wafers modified with (3-aminopropyl)triethoxysilane (APTES). The reaction between imidazole derivatives introduced to the silicon wafers with hydroxyl groups contained in the P(DMAEMA-co-OEGMA-OH) star arms was used for polymer immobilization. Such an approach was studied in detail in our previous work, ensuring stable covalent attachment of stars to the chemically modified quartz crystal microbalance sensors [22].

**Figure 5:**
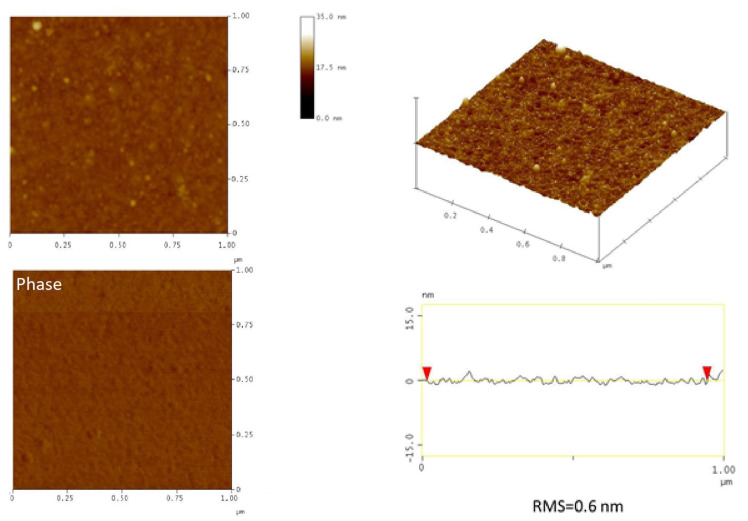
AFM images of P(DMAEMA-co-OEGMA-OH) star layers (sample SC-L).

The physico-chemical characterization of the obtained star P(DMAEMA-co-OEGMA-OH) layer (sample SC-L) was performed inter alia using atomic force microscopy (AFM), ellipsometry and X-ray photoelectron spectroscopy (XPS). The obtained layers were relatively thin, their thickness, measured ellipsometrically, was equal to 8 ± 1 nm. Based on the results obtained from contact angle measurements, it could be concluded that the layer is also moderately hydrophilic, as its measured value was equal to 62 ± 2°. The AFM phase images showed that the silicon wafers before and after reaction of quaternization were completely covered with star polymer, but the surface morphology was rough (Figure 5 and Figure S1, Supporting Materials). The average roughness (RMS) of the layer before quaternization calculated based on AFM images was equal to 0.6 nm (Figure 5). The self-assembly of stars present in the solution (confirmed by DLS) may affect the roughness of the polymer layers. The properties of obtained layers on the silicon wafers were similar to those prepared on sensors used in crystal quartz microbalance technique [22]. 

In the next step, the P(DMAEMA-co-OEGMA-OH) star layers were quaternized with bromoethane (sample SC-LQ_2_) and 1-bromooctane (sample SC-LQ_8_). The applied conditions were based upon those established for reaction performed earlier in the solution, therefore it was also checked in the case of 1-bromooctane, whether extending the reaction time may enhance the obtained quantity of ammonium groups in the obtained layers.

The quantity of ammonium groups in the star copolymer layer was measured using UV-Vis spectroscopy by measuring the amount of bonded fluorescein to the ammonium groups, according to the procedure described in the literature [29,30]. In the measurements, it was assumed that one fluorescein molecule corresponds to one quaternary group of the attached polymer (the dye binds only to ammonium groups). The mass of fluorescein sodium salt eluted from 1 cm^2^ of surface was directly measured and then recalculated to the amount of ammonium groups per 1 cm^2^ of surface.

The values of the quantity of ammonium groups introduced to P(DMAEMA-co-OEGMA-OH) layers for both ethyl and octyl bromide are similar (Table 1).

In the case of the layer SC-LQ_8_, the calculated mass of fluorescein sodium salt after washing from 1 cm^2^ of surface quantity of quaternized groups, measured after 7 days were 1.98∙10^−4^ g and 3.17∙10^17^, respectively, and did not differ significantly from the values obtained after 24 h, which were within the error range (Table 1). Thus, the extended reaction time, as was the case for the star copolymer in solution, had no significant effect on the efficiency of the reaction.

The obtained values of the quantity of ammonium groups introduced after quaternization with bromoethane for the layers of P(DMAEMA-co-OEGMA-OH) are an order of magnitude smaller than for the layers of homopolymer DMAEMA stars, obtained in our group [19]. This is probably caused by the method of attachment of star polymers to the surface. In our previous work, PDMAEMA stars were immobilized using photochemical reactions, which led to the formation of multilayers of much greater thickness in comparison with thin nanolayer obtained here, and therefore a significantly higher number of ammonium groups [19]. Furthermore, the presence of OEGMA-OH in the structure of the star polymer may affect the quaternization reaction, probably hindering the access to amino groups. This is due to both the complex structure of the monomer itself and the method of copolymerization (OEGMA-OH is mainly incorporated in the ends of the star polymer arms). To summarize, analogously to the solution reaction, the quantities of ammonium groups in the case of the 1-bromooctane are slightly lower than those obtained for the shorter one, which may indicate worse availability of amino groups for longer alkyl chains.

The introduction of alkyl chains into the structure of stars also had an effect on the affinity to water of such layers. Compared to the unmodified layers, the contact angle values increased by almost 10 degrees in the case of the layer SC-LQ_2_, (ϴ = 71 ± 3°) and by as much as 22 degrees for the layer SC-LQ_8_ (ϴ = 84 ± 3°). The increase in the hydrophobicity of quaternized nanolayers also indirectly proves the efficient modification of the layers.

**Figure 6:**
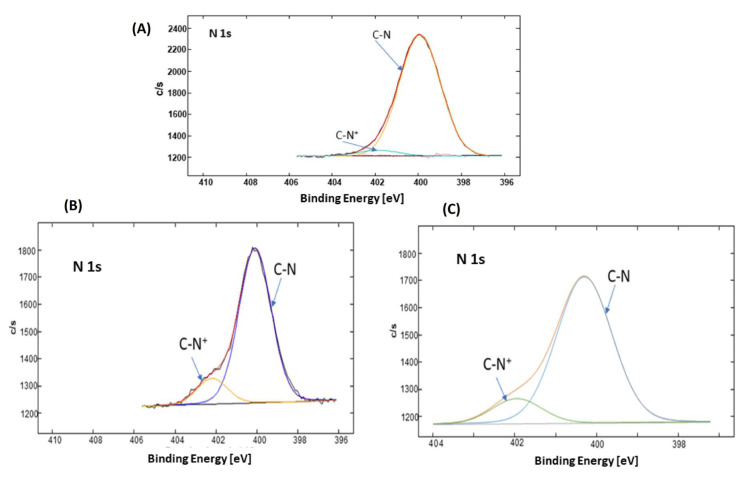
The N 1s core level XPS spectra of the star copolymer layers (**A**) before quaternization (sample SC-L) and (**B**) after quaternization with bromoethane (sample SC-LQ_2_) and (**C**) after quaternization with 1-bromooctane (sample SC-LQ_8_).

The presence of quaternized groups in the P(DMAEMA-co-OEGMA-OH) layers was confirmed by XPS. Figure 6 shows the high-resolution N 1s spectra before (Figure 6A) and after the quaternization reaction (Figure 6B,C) of P(DMAEMA-co-OEGMA-OH) layers.

In the high-resolution N 1s spectra (Figure 6B,C), an increase in the C-N^+^ signal was observed in comparison to the spectrum before the quaternization reaction (Figure 6A). The relative percentage of C-N and C-N^+^ bonds before and after quaternization is shown in Table S1 (Supporting Materials). However, this increase was not as significant as in the case of star homopolymer layers quaternized with ethyl bromide [19]. The reason for this is probably also connected with layer thickness and number of star shaped macromolecules on the surface.

### 3.3. Evaluation of Antibacterial Activity of the Star Nanolayers

The effect of alkyl chain length of a quaternary agent on the antibacterial properties of studied polymers varies greatly. Some of the authors describe an increase in biocidal properties with an increase in the length of the alkyl chain [6,16], while others observe the opposite effect [14,30,31]. Our previous studies showed that a star with homopolymer arms of DMAEMA with Mn = 115,000 g/mol quaternized with ethyl bromide exhibited higher antibacterial activity to both Gram-positive (*B. subtilis*) and Gram-negative (*E. coli* and *P. aeruginosa*) bacterial strains than the analogous linear polymer [26]. Therefore, the performed research became the starting point for exploring the possibility of direct quaternization of polymer layers made of stars.

Here, we studied the influence of two alkyl bromides: bromoethane and 1-bromooctane on the biocidal properties of the P(DMAEMA-co-OEGMA-OH) layers (SC-LQ_2_ and SC-LQ_8_, respectively). Inhibition of the bacterial growth on unmodified P(DMAEMA-co-OEGMA-OH) layers (sample SC-L) was also tested. The antimicrobial potential of all tested layers was tested against the model strains of Gram (+) *Bacillus subtilis* and Gram (−) and *Escherichia coli* which are dangerous pathogens for human health. Unmodified silicon substrates were used again as a control. Inhibition of bacterial growth was examined after 1 h and after 24 h contact with the nanolayers (Table 2).

The highest inhibition of bacterial growth after 1 h of exposure was observed for copolymer layers quaternized with 1-bromooctane (SC-LQ_8_) for both tested bacterial strains. Similar results were obtained for PDMAEMA grafted onto poly(p-chloromethylstyrene) microspheres [16]. The authors observed that with the increase in the number of methylene groups in the chain of the alkyl halide used for quaternization, the activity of the system against *E. coli* and *S. aureus* strains increases, due to the increase in the hydrophobic properties of the material [16]. In the studies for the linear polymer DMAEMA grafted on cellulose against *E. coli* the higher alkyl bromides (C8, C12 and C16) were investigated as quaternizing agents [14]. Similar to the results obtained in this work, the best antibacterial properties exhibited surfaces quaternized with 1-bromooctane [14].

Surprisingly, after 24 h of contact with the bacterial culture, an increase in antibacterial activity was observed for the polymer layers modified with shorter alkyl chains. Inhibition of bacterial growth was also strongly dependent on the type of bacterial strain. The best growth of both *B. subtilis* and *E. coli* was inhibited (100% after 24 h) by bromoethane quaternized layers (SC-LQ_2_). Additionally, unmodified layers (SC-L) showed high levels of growth inhibition of 95% to *E. coli*. Similar results were observed for linear PDMAEMA grafted on the cellulose against *E. coli* [14].

Based on the results of the performed tests, it can be concluded that the layers quaternized with 1-bromooctane exhibit the best antibacterial properties after a short exposure time, while layers quaternized with bromide containing a shorter aliphatic chain works better after a longer contact time with the bacterial strains.

## 4. Conclusions

The star polymers of DMAEMA and OEGMA-OH obtained via ATRP were quaternized in the solution and after formation of covalently bonded layers with silicon wafers. Two alkyl bromides, with various methylene units were used for this purpose: bromoethane and 1-bromooctane. The results showed that the yield of the quaternization reaction is dependent on the length of the alkyl chain in the bromide, the conformation and self-assembly process of the stars in the solution and significantly decreases when a bromide with a longer alkyl chain is used. In the case of the star quaternization process carried out for macromolecules in the form of nanolayers covalently bound to a solid substrate, it seems that the influence of the alkyl agent used does not significantly determine the efficiency of the process. This allows us to conclude that it is better to carry out modifications of such polymeric nanostructures after and not before their attachment to the surface. Obtained nanolayers exhibited biocidal effects for all studied bacterial strains. It was shown that along with the extension of the contact time of the tested nanolayers with bacteria, the change of the length of alkyl chain in the used bromide from C8 to C2 increased the antibacterial activity of the entire system. The obtained results are important and novel from the point of view of examining how the structure of such layers affects the potential use of them to destroy various strains of bacteria, which is detrimental to obtaining effective polymeric biocidal materials for use in medicine.

## Figures and Tables

**Table 1:** Mass of fluorescein sodium salt eluted from 1 cm^2^ of surface and quantity of ammonium groups introduced to P(DMAEMA-co-OEGMA-OH) star layers.

Layer	Mass of Fluorescein Sodium Salt Eluted from 1 cm^2^ of Surface [g]	Quantity of Ammonium Groups on cm^2^ of the Surface
SC-LQ_2_	2.79∙10^−4^	4.47∙10^17^
SC-LQ_8_	2.22∙10^−4^	3.55∙10^17^

**Table 2:** Growth inhibition of *Bacillus subtilis* and *Escherichia coli* by star copolymer layers after 1 and 24 h of exposure.

Strain	Sample	Number of Bacteria [CFU/mL] 1 h	Growth Inhibition * [%] 1 h	Number of Bacteria [CFU/mL] 24 h	Growth Inhibition * [%] 24 h
*E. coli*	SC-L	2.0∙10^5^	41	1.4∙10^4^	95
	Control	3.4∙10^5^	–	3.2∙10^5^	–
	SC-LQ_2_	1.4∙10^8^	30	0	100
	Control	2.0∙10^8^	–	2.0∙10^8^	–
	SC-LQ_8_	2.6∙10^4^	92	6.8∙10^4^	79
	Control	3.4∙10^5^	–	3.2∙10^5^	–
*B. subtilis*	SC-L	2∙10^4^	90	3.3∙10^5^	51
	Control	2∙10^5^	–	6.7∙10^5^	–
	SC-LQ_2_	2.67∙10^3^	11	0	100
	Control	3.0∙10^3^	–	2.42∙10^3^	–
	SC-LQ_8_	2.2∙10^4^	89	3.2∙10^5^	52
	Control	2∙10^5^	–	6.7∙10^5^	–

* The percentage of growth inhibition was calculated relative to the control after a given time.

## Data Availability

The data presented in this study are available on request from the corresponding author.

## Supplementary Materials

The following supporting information can be downloaded at: https://www.mdpi.com/article/10.3390/polym15051260/s1, Table S1. Relative percentage of C-N and C-N+ bonds of P(DMAEMA-co-OEGMA-OH) star layers determined by XPS technique. Figure S1. The AFM phase images of P(DMAEMA-co-OEGMA-OH) star layers after quaternization: (**A**) with bromoethane (sample SC-LQ2) and (**B**) with 1-bromooctane (sample SC-LQ8).
