# Supplementary material for: Quaternized Poly(N,N′-dimethylaminoethyl methacrylate) Star Nanostructures in the Solution and on the Surface
Source: Polymers (Basel). 2023 Mar 1;15(5):1260. doi: 10.3390/polym15051260 (PMC10007306; doi:10.3390/polym15051260)
Supplement: Supplementary file 1 [file polymers-15-01260-s001.zip › polymers-2229683-supplementary.pdf]

Supplementary Materials:

Quaternized Poly(*N,N′*-dimethylaminoethyl methacrylate) Star Nanostructures in the Solution and on the Surface

Paulina Teper, Anna Celny, Agnieszka Kowalczuk and Barbara Mendrek *

Centre of Polymer and Carbon Materials, Polish Academy of Sciences, M. Curie-Sklodowskiej 34, 41-819 Zabrze, Poland
* Correspondence: bmendrek@cmpw-pan.pl

Table S1. Relative percentage of C-N and C-N$^+$ bonds of P(DMAEMA-co-OEGMA-OH) star layers determined by XPS technique.

Relative percentage of bond [%]	Sample SC-L	Sample SC-LQ$_2$	Sample SC-LQ$_8$
**C-N**	95.2	89.5	88.6
**C-N$^+$**	4.8	10.5	11.4

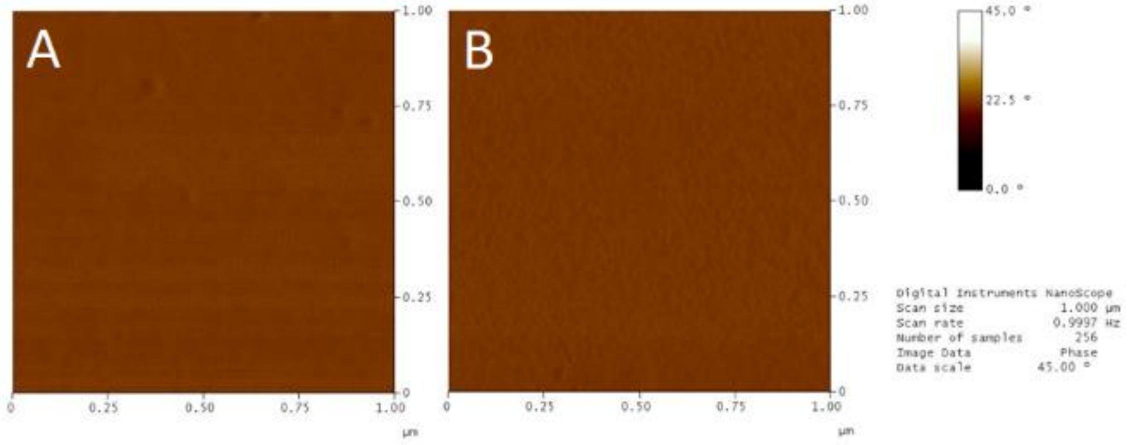

Figure S1. The AFM phase images of P(DMAEMA-co-OEGMA-OH) star layers after quaternization: (**A**) with bromoethane (sample SC-LQ$_2$) and (**B**) with 1-bromooctane (sample SC-LQ$_8$).
